# Recent Advancements in Information Ratchet Design

**DOI:** 10.3390/molecules31081282

**Published:** 2026-04-14

**Authors:** Sara Incarbone, Luca De Gioia

**Affiliations:** Department of Biotechnology and Biosciences, University of Milan-Bicocca, Piazza della Scienza 2, 20126 Milan, Italy; luca.degioia@unimib.it

**Keywords:** information ratchet, energy converter, energy dissipation, endergonic synthesis, biomimetics, carbodiimide

## Abstract

While the broader context of molecular machinery has already been extensively discussed in the scientific literature, there is a lack of dedicated reviews focusing specifically and exclusively on information ratchets. These ratchets deserve a dedicated analysis as they are common in nature and their implementation in artificial systems can lead to new ways of achieving biomimetic processes and endergonic synthesis. This review summarizes recent advancements in the design of synthetic information ratchets, highlighting breakthroughs in the rationalization and optimization of fueling and structural parameters for the sake of efficiency. Novel methods are described for in situ quantification and the translation of molecular motion into macroscopic work. The latest artificial information ratchets are compared to the previous literature and the natural motors that inspired them. The reported findings are meant to show how research on information ratcheting has progressed in the last five years, with various designs paving the way to bio-inspired nanotechnologies and materials.

## 1. Introduction

Endergonic processes, despite lacking spontaneity, are fundamental in biology as nature manages to transduce energy in order to power thermodynamically unfavorable outcomes that would not be achievable otherwise [1,2,3,4]. For example, membrane proteins can turn ADP and Pi into ATP by using light or chemical compounds as external sources of energy and converting such energy into electrochemical potential [5]. In order to carry out endergonic synthesis, an energy source is not enough; it is necessary to rely on a globally exergonic mechanism that couples an exergonic process with an endergonic one, such that the free energy of the former can be harnessed and used to drive the latter [6]. At the molecular scale, biological “engines” are enzymes that couple thermodynamically spontaneous processes with endergonic reactions, such that both reactions can reach completion [7,8]. Enzymes catalyze the individual steps of a chemical process, allowing reactions to occur fast enough that life can sustain itself. Enzymes are very powerful as they can speed up reactions well over a million-fold in magnitude [9]. Furthermore, they can be highly specialized in catalyzing specific types of molecules into new ones. Enzymes can even be extracted and used to catalyze commercially important processes for industrial applications, such as the modification of antibiotics and the production of sweetening agents, washing powders and more [10].

Some enzymes are not just biological catalysts; they can also function as free-energy converters, capable of transducing the free energy from external fluctuations in their environment [11]. For many years, chemists have taken an interest in developing artificial systems that mimic the efficiency found in biology, thus aiming to design synthetic motors that can harvest energy away from equilibrium in order to perform work [1,12,13]. There is quite a large variety of molecular motors, both natural and synthetic, that drive molecular systems away from equilibrium and introduce a directional bias that leads to desired outcomes [2,14]. These molecular motors are also described as ratchets [1,14]. Ratchets rely on kinetic asymmetry, first described by Astumian et al. in 1989 [11]. Kinetic asymmetry is the key factor that determines whether a ratchet can use energy-driven fluctuation to create order and generate useful output work in a given system. Kinetic asymmetry is the modulation of kinetic barriers that, when coupled to an energy input, leads to a kinetic bias in favor of one cycling direction over the other in a chemomechanical cycle, thus establishing directionality [14]. The ratcheting constant, K_r_, can be used to quantify directionality because it identifies whether a system prefers to cycle in the clockwise or counterclockwise direction [3]. Ratcheting in coupling systems uses input energy to constrain random motion in favor of the desired pathways while preventing unproductive motion that may hinder the system’s ability to generate the desired products [15]. There are different types of energy input: some ratchets are light-driven while others are driven by catalysis [2,16]. Ratchets can also differ from one another based on the strength of their coupling: ratchets can feature strong or weak coupling. Strong coupling occurs if the flux through the forward reverse cycle is much greater than the flux through the backward cycle, such that applying a sufficiently high force or torque causes the motor to transduce mechanical energy to chemical energy by converting waste to fuel when back driven by torque. Strong or weak coupling is determined by the relative magnitudes of the chemical potential difference of the driving reaction and the kinetic asymmetry [17]. The majority of biological molecular motors in nature (e.g., kinesin, myosin, dynein) are weakly coupled [12]. In addition, coupling can be described as either tight or non-tight. Both cases exist in nature. For instance, a hexameric helicase is a motor that shows tight or nearly tight coupling under hindering external forces, whereas monomeric helicases, kinesin, and dynein feature non-tight chemomechanical coupling, meaning that their activity is independent or nearly independent of the external forces acting on them [18]. Another distinction worth mentioning is the one that differentiates between energy ratchets and information ratchets. Energy ratchets rely on external modulation with an energy supply that changes over time or space [1,19]. Conversely, information ratchets do not require variation of external conditions; they perform work as long as there is fuel available [1,19]. Ratchets rely on kinetic asymmetry and, in the case of an information ratchet, the ratchet mechanism rectifies the direction of a stochastic process to kinetically discriminate between the rates of formation and depletion of species within a reaction network, creating a directional bias in favor of the kinetically preferred cyclic pathway under non-equilibrium conditions [20]. The energy barriers of the reaction steps act as a series of gates [21], hence the directional bias in information ratcheting is a matter of controlling the energy barriers of the reaction steps [22]. The majority of molecular motors found in biology are considered to function as information ratchets [1,19,20,23]. This review specifically focuses on information ratchets, due to their relevance in biology and their potential for future bio-inspired applications. Moreover, this review highlights the most recent findings regarding the designs and applications of information ratchets that may narrow the gap between man-made molecular systems and nature’s machinery.

## 2. Information Ratchets

### 2.1. A Fast Record-Breaking Dual-Motor

**Figure 1:**
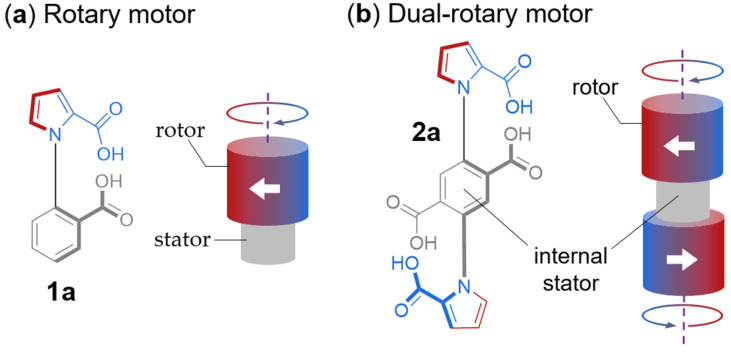
(**a**) Chemical structure and schematic representation of the single-motor molecule (**1a**) [25]. (**b**) Chemical structure and schematic representation of the dual rotary motor molecule (**2a**). The dual-motor is characterized by S_2_ symmetry, resulting in coaxial rotation of the rotors in opposite directions [24].

Borsley et al. designed and tested a dual-motor for a system that originally featured a single-motor and demonstrated how the structure of the dual-motor substantially improved the speed of the chemically fueled rotation [24]. The motor consists of two identical motor units whose pyrrole-2-carboxylic rings are turned in a disrotatory fashion around a shared phenyl-2,5-dicarboxylic acid stator. Just like its parent single-motor analog (Figure 1a) [25], the dual-motor (Figure 1b) directionally rotates via an information ratchet mechanism.

**Figure 2:**
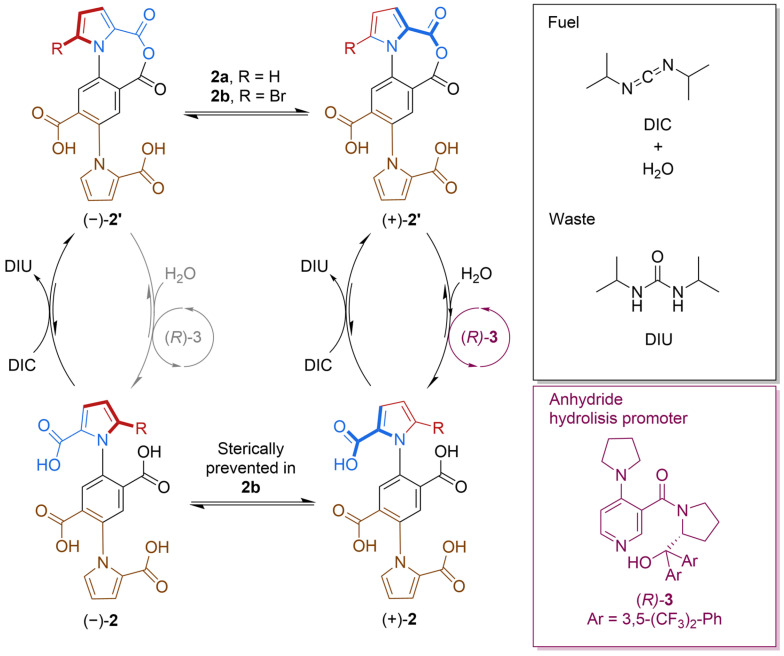
The chemomechanical cycle of the dual-motor, featuring the catalysis-driven rotation of the upper pyrrole ring (the top rotor; red-blue). The lower motor unit (brown) operates through an identical mechanism. The fuel is diisopropylcarbodiimide (DIC) and the resulting waste is diisopropylurea (DIU).

The mechanism (Figure 2) is powered by the hydration of a carbodiimide, which forms urea as waste.

The fuel-to-waste reaction is catalyzed through the chemomechanical cycle of a motor unit, causing directional rotation about a biaryl C–N bond. This chemically fueled information ratchet was able to perform a continuous contrarotation on a time scale of 2–4 min per rotation. The dual-motor proved to be approximately seven times faster than the parent 1-phenylpyrrole-2,2dicarboxylic acid single-motor when operated under identical conditions, and 90 times faster than the single-motor when operated using the originally reported reaction conditions [25]. Motor 2b rotated with a modest directionality of 1.5:1 (i.e., a backward rotation every ∼2.5 forward turns). Relying on a chiral anhydride hydrolysis promoter caused preferential hydrolysis from one face of the motor with good enantioselectivity (2.3:1), leading to a significant kinetic asymmetry (K_r_ = 1.5) in the chemomechanical cycle. Although the dual-motor’s rotation is still orders of magnitude slower than the one of biomolecular motors, it is the fastest sustained speed of rotation measured to date for artificial small-molecule motors. These results showed how the inclusion of two cooperative motor units can offer better rotation. This design is promising for future nanoscale mechanical applications.

### 2.2. Structural Influence of the Chemical Fueling System on a Catalysis-Driven Rotary Motor

**Figure 3:**
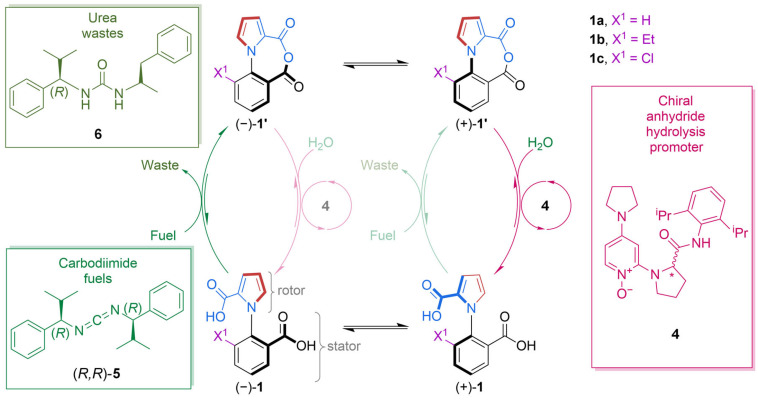
The chemomechanical cycle of the diacid motor molecule and the structures of the fuels and the hydrolisis promoter.

Liu et al. worked on optimizing the single-motor originally designed by Borsely et al. [25], as shown earlier in Figure 1a. This ratcheting system consists of a chemically fueled 1-phenylpyrrole 2,2′-dicarboxylic acid rotary molecular motor [26]. The directional 360° rotation of the pyrrole rotor around the phenyl stator was coupled with the motor’s catalysis of carbodiimide hydration (Figure 3).

Kinetic asymmetry was exclusively determined via chemical gating based on the kinetically controlled enantioselectivity of anhydride formation (Figure 1, dark green vs. light green) and anhydride hydrolysis (Figure 1, dark pink vs. light pink). The authors considered the possible coupled cycles (productive pathways), futile cycles (fuel consumption without net movement of the motor), and step cycles (movement without net consumption of fuel, occurring with the same frequency in both the clockwise and counterclockwise directions in the absence of an applied force, decreasing directionality). Slip cycles did not affect the motor’s efficiency because they were extremely rare. The rarity of the slip cycles was due to the fact that the network featured a large activation energy barrier for the acid groups to slip past each other in the diacid state of the motor, and the acyl groups could not pass each other (except through ring-flipping) in the anhydride state of the motor. Clockwise coupled cycles were favored by perfecting the degree of chemical gating, which in turn increased kinetic asymmetry. Considering that the two chemical gatings could combine multiplicatively to generate the overall kinetic asymmetry, small changes were sufficient for the improvement in the motor’s overall directionality. The stereochemistry of the carbodiimide fuel only affects anhydride formation and the stereochemistry of the hydrolysis promoter only affects anhydride hydrolysis. It was therefore possible to independently optimize the different reagents in the chemical fueling system for the chemical step they perform in the catalytic cycle. Different chiral substituents were used for the fuel and chiral promoter, some aliphatic and some aromatic, providing variety in terms of sterics and stereoeletronic characteristics. After testing the motor’s performance by varying the structures of the carbodiimide fuel and the anhydridre hydrolysis promoter, the authors concluded that the system was optimized when using N,N-di(isopropylbenzyl)carbodiimide ((*R*,*R*)-**5** in Figure 3) and N-oxide anhydride hydrolysis promoter (**4** in Figure 3). Combining the best-performing hydrolysis promoter and fuel ((*R*)-**4** and (*R*,*R*)-**5**) into a single fueling system resulted in a 92% enantiomeric excess with a directionality of ∼24:1 in favor of the enantiomer (−)-**1b**, a remarkable increase compared to the ∼3:1 directionality of the original fueling system [25]. However, such efficiency and high directionality came with a price: a slow rate of fuel addition. The slow rate of fuel addition led to a modest speed of rotation (each catalytic cycle took ∼40 h under these conditions). This kind of trade-off is common when designing artificial molecular motors and macro-scale motors, and it is even observable in biomolecular machinery. Indeed, it is quite difficult for a motor to be both directionally selective and fast at the same time. Usually, certain conditions improve one attribute at the expense of another, as explained in detail by Borsley et al. in a previous article, which also dealt with a chemically fueled information ratchet involving carbodiimide fuel [27]. Overall, the performance of the minimalist catalysis-driven molecular motor reported by Liu et al. seemed to be closely related to the transduction of chemical energy that is ubiquitous of motor catalysis in biology.

### 2.3. Gel Expansion and Contraction

**Figure 4:**
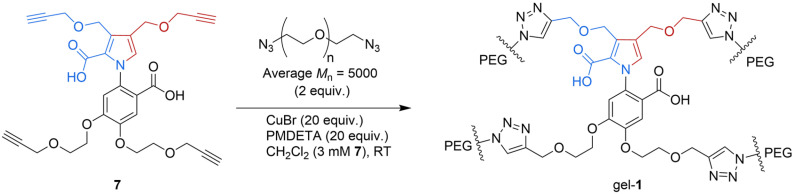
Tetra-alkyne **7** was treated with bisazide-terminated polyethylene glycol (PEG; number-average molecular weight: M_n_ = 5000 g mol^−1^), CuBr and N,N,N′,N″,N″-pentamethyldiethylenetriamine (PMDETA) in CH_2_Cl_2_. Thanks to the copper(II)-catalyzed azide-alkyne cycloaddition (CuAAC), the motor was incorporated into a cross-linked gel (gel-**1**) through the chemical cross-linking of polymer chains at the motor nodes. RT, room temperature.

A cross-linked polymer gel was prepared by Wang et al. such that it could contract and re-expand, thanks to the directional rotation of artificial catalysis-driven molecular motors [28]. The gel’s behavior is the result of the molecular-level transduction of chemical energy to mechanical force. Specifically, the polymer chains (Figure 4) of the cross-linked network are twisted due to the continuous 360° rotation of the rotor about the stator of the catalysis-driven motor-molecules.

**Figure 5:**
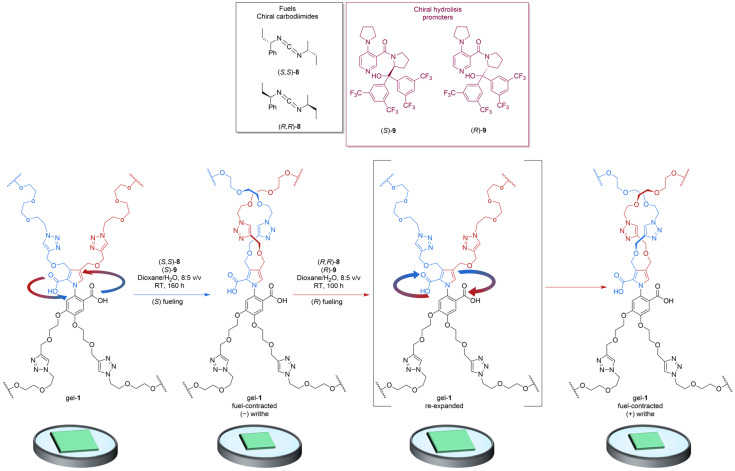
Chemically fueled expansion and re-contraction of a sample of gel-**1**. A gel-**1** sample is contracted with (*S*,*S*)-**8** and (*S*)-**9** (anticlockwise rotation) and then exhaustively washed to remove waste, residual fuel, and hydrolysis promoter. The gel sample is then treated with (*R*,*R*)-**8** and (*R*)-**9** (the fueling system of opposite chirality) to power the rotation of the motor components in the opposite direction (clockwise). The second fueling system first powers the untwisting of the anticlockwise-twisted polymer strands, causing a re-expansion of the previously contracted gel, then the catalysis-driven rotation begins to reintroduce writhe of the opposite twist sense, leading to a re-contraction.

A thin square of gel was prepared with the following dimensions: 10 × 10 × 1 mm^3^, around 0.08 mmol motor units. This gel sample was treated with a solution of the chiral hydrolysis promoter in dioxane/H_2_O at room temperature. The increasing entanglements of the gel reduced its volume by approximately 30%. The contraction was reversible by using a fuel system of opposite chirality, which powered the motors to rotate in the reverse direction, causing the unwinding of the polymer chains and the consequent re-expanding the gel. After re-expansion with the use of (*R*,*R*)-**8** and (*R*)-**9**, the rotation of the motors under catalysis continues in the clockwise direction and the gel starts to re-contract, reaching a minimum volume of 62% after approximately 60 h of fueling. (Figure 5).

Kinetic asymmetry is ensured by the motor-molecule units in the gel, which generate force by fueling the biasing of the kinetics of ground-state conformational changes. The motor-molecule units in the gel act through a catalysis-driven information ratchet mechanism that is typical in motor proteins, such as myosin II. This experiment differs from other artificial chemically responsive gels, which usually operate through switching (and therefore operate as energy ratchets rather than information ratchets).

### 2.4. Abiotic Example of an Information Ratchet That Improves Selectivity in Molecular Recognition

**Figure 6:**
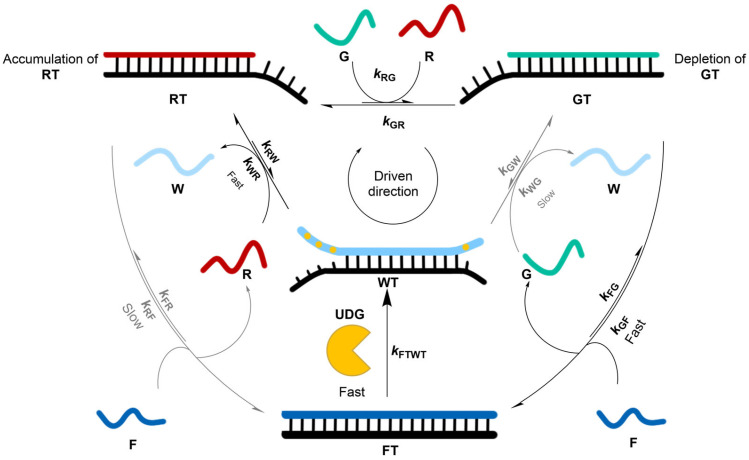
A schematic representation of the network in which a fuel-to-waste reaction (F: fuel strand, W: waste) can drive DNA duplex exchange away from equilibrium, thus increasing the proportion of the red RT duplex. The black arrows indicate the kinetically preferred pathway for the reaction network. The enzyme, uracil-DNA glycosylase (UDG), converts FT into WT, weakening the association with T by removing uracil residues from the F strand. W is then preferentially displaced by R or G, with a kinetic bias toward a faster reaction with R due to a larger toehold. The short DNA sequences of the reacting species (R, G, T and F) are the source of kinetic asymmetry for the overall reaction network.

Roberts et al. recently published a computational analysis of a proposed model of DNA hybridization to show how an information ratchet mechanism (Figure 6) increases the selectivity of molecular recognition [20].

The model successfully selected the correct duplex from 2:1 at equilibrium to 6:1 under energy-dissipating conditions. Kinetic asymmetry, on which an information ratchet depends, was introduced by the structural asymmetry in the DNA strands. Roberts et al. argued that molecular recognition based on thermodynamic selectivity alone is not enough for life to persist. Instead, biomolecular machines perform error correction by kinetic proofreading, thus ensuring sequence-specific synthesis with higher fidelity. The greater selectivity is made possible through energy consumption, meaning that kinetic proofreading functions as an information ratchet mechanism, which is kinetically selective and makes it possible to fuel an endergonic process via energy dissipation. The authors designed a minimalistic reaction network that transduces energy from a fuel-to-waste reaction that enables enhanced selectivity during competitive DNA hybridization. The improved selectivity was determined by the thermodynamics of binding and the kinetic asymmetry within the network. The model was tested and analyzed by running simulations, which showed the change in the template’s preferences for binding partners while consuming free energy. The model proved that kinetic selection results from the inherent structures and reactivities of the species involved in the network through an information ratchet mechanism. The design of this model suggests that information ratchet mechanisms can have general applications for error correction in both abiotic and biotic contexts. The authors concluded that information ratchet mechanisms are both necessary and sufficient for error correction and that they have the potential for the development of more effective chemical sensors and dissipative DNA technologies. Another problem addressed was the high background rate of fuel consumption, typical in synthetic non-equilibrium systems. However, the authors suggested that this setback can be overcome by using fuels that are only activated when bound, such that they are only consumed by the ratchet mechanism, making the overall catalytic cycle more efficient.

### 2.5. In Situ Quantification of Directional Rotation by a Catalysis-Driven Acid

**Figure 7:**
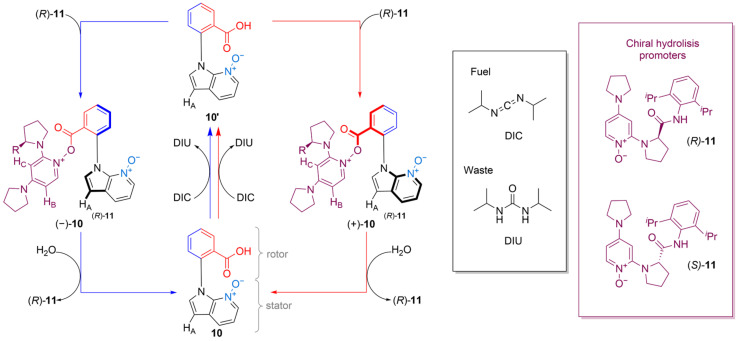
A schematic representation the cycle of motor **10** and the structures of the fuels and hydrolysis promoters. In the presence of achiral pyrrolidinylpyridine N-oxide, the catalysis via carbodiimide hydration—diisopropylcarbodiimide (DIC) to diisopropylurea (DIU)—allows the motor to undergo directional rotation, either clockwise (red cycle) or counterclockwise (blue cycle).

Liu et al. reported a new class of artificial chemically fueled molecular motor, featuring a phenyl carboxylic acid rotor attached to a 7-azaindole-N-oxide stator through an abiaryl C–N bond [29]. The motor’s directional rotation was quantified in situ. The catalysis-driven reaction that provided fuel was, once again, carbodiimide hydration. The carbodiimide hydration occurs in the presence of a chiral pyrrolidinylpyridine-N-oxide. The catalytic cycle (Figure 7) features an intermediate O-acyl-azaindole-N-oxide ester tether that forms between the carboxylic acid of the rotor and the N-oxide of the stator.

Compared to previously reported catalysis-driven motors, this ratchet’s directionality could be determined directly from the transient concentrations of the diastereomeric intermediates formed during rotary catalysis. This approach provided direct access to other key performance indicators, including motor speed and catalytic, coupling, and fuel efficiency. The real-time in situ quantification measurements made it possible to determine the kinetic asymmetry of the ratchet (motor 10 in Figure 7), with the following value for the ratcheting constant: K_r_ = 2.1. The results demonstrated that this novel and straightforward method for in situ quantification of motor behavior can help with the design of new ratchets, the rationalization of their attributes, and their optimization.

### 2.6. An Information Ratchet with a Cone-Shaped Macrocycle

**Figure 8:**
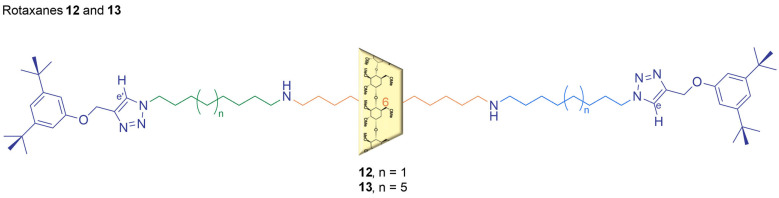
A schematic representation of the structure of rotaxane **12** and **13**, featuring the surrounding 3D macrocycle, alpha-cyclodextrin.

Information ratchets usually modify the energy landscape of a system based on the relative position of the motor components. However, Liu et al. designed a new type of information ratchet that relies on the shape of a cyclodextrin macrocycle: in this system, both the shape and orientation of a 3D macrocycle provide dual input for ratcheting (Figure 8) [30].

**Figure 9:**
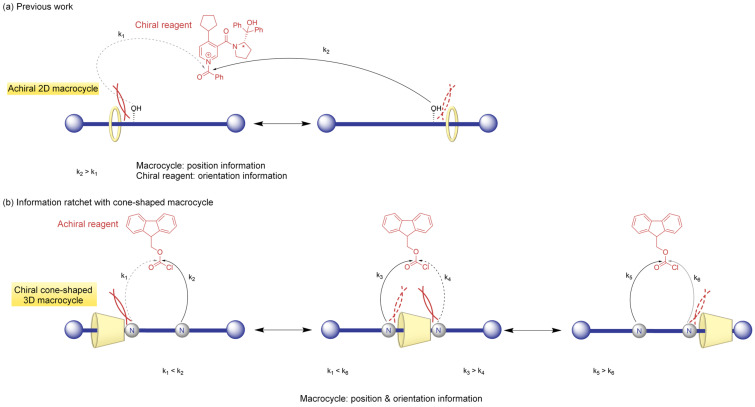
(**a**) The information ratchet designed by Leigh’s group. The ratcheting mechanism is based on the position of a macrocycle on a prochiral axle reacting with a chiral reagent [31]. (**b**) Information ratchet developed by Liu et al. The ratcheting mechanism is based on the position and orientation of a cone-shaped macrocycle (cyclodextrin) threaded onto a achiral axle reacting with an achiral reagent [30].

Liu et al. took inspiration from an information ratchet designed and studied by Leigh’s group, which featured a 2D macrocycle on an axle. Leigh’s system was based on a rotaxane threaded on a prochiral axle, with two equivalent stations separated by a prochiral hydroxyl group that functioned as an information ratchet when reacting with an anhydride and a chiral base in the presence of the macrocycle [31]. For this 2D macrocycle system, the rate of the reaction was only sensitive to the position of the achiral macrocycle on the axle, considering that the direction of approach of the anhydride toward the hydroxyl group on the axle was controlled by the base’s chirality. Both Leigh’s group and Liu et al. used a rotaxane as the ratcheting component for their respective systems. However, Liu et al. used a 3D macrocycle instead of a 2D one (Figure 9).

A permethlyated alpha-cyclodextrin (α-CD) was chosen as the 3D macrocycle as it is a natural, readily available and cheap cyclic chiral carbohydrate that adopts a conical shape. Thus, the chiral 3D macrocycle presented two different faces to the reactive center. Two different versions of the same system were studied by Liu et al., using two rotaxanes of different lengths. The rotaxanes consisted of two stoppers and three alkyl segments separated by two secondary amines, which reacted with an achiral reagent. Additionally, 9-fluorenylmethyloxycarbonyl (Fmoc) protecting groups were introduced to protect the amines of the rotaxanes and add steric barriers between the three segments. The kinetic biases were assessed through the analysis of product ratios, and the results showed that Fmoc protection was 3-fold faster when the cyclodextrin’s larger rim was facing toward the amine group. This proved that ratcheting depended on two sources of information: the position and the orientation of the cone-shaped cyclodextrin relative to the rotaxane. This study showed how the conical shape of cyclodextrins can lead to an information ratchet with double input. Moreover, the application of cyclodextrins opens the possibility of selective face functionalization that can improve ratchet efficiency and offer new ratchet designs, such as the use of water-soluble cyclodextrins for information ratcheting in aqueous media.

**Figure 10:**
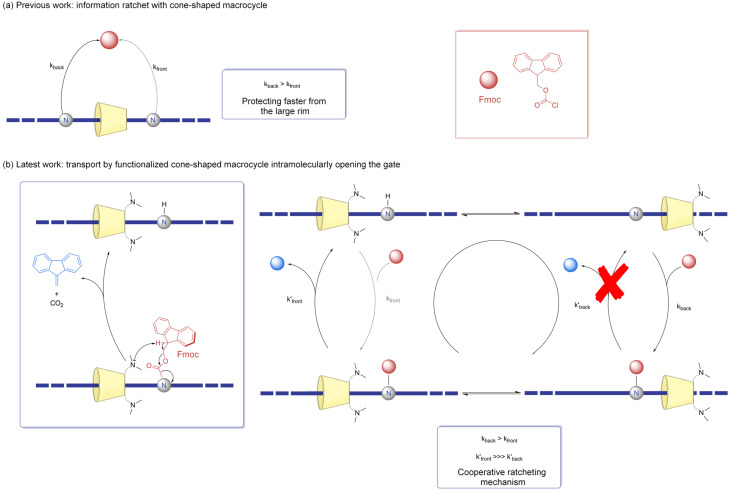
(**a**) The information ratchet designed by Liu et al. in 2023, as seen in Figure 9 [30]. (**b**) The latest information ratchet developed by Liu et al. in 2025, achieving unidirectional transportation with a cyclodextrin that is functionalized by tertiary amines [32].

Liu et al. studied the ratcheting system further by attaching two tertiary amine groups to the primary (smaller) rim of the 3D macrocycle, leading to an innovative one-way ratchet mechanism (Figure 10) that operates irreversibly and is driven by chemical fuel [32].

The tertiary amines were chosen because the previous literature reported that they can slowly deprotect Fmoc groups [33]. The rotaxane still featured amines near the ends of the axle, which were protected by Fmoc groups in order to focus on the effect of the additional tertiary amines on the small rim. The tertiary amines were studied to evaluate whether they could induce a regioselective intramolecular deprotection. Grafting the amines directly onto the α-CD appeared to hinder its ability to deprotect the Fmoc groups, but this issue was overcome by linking the amines and the cyclodextrin via a two-carbon spacer. The 3D structure of the α-CD was responsible for regioselectivity, while the grafted tertiary amines on the small rim enabled mechanospecific deprotection of the Fmoc groups on an amine located on the axle in front of the rim’s smaller face. The rim-selective functionalization and the deprotection reaction of Fmoc groups resulted in a double-gated ratchet, exploiting a combination of kinetic biases to enable unidirectional movement of the cyclodextrin along the axle. Thanks to the inherent chirality of cyclodextrins, the directionality information was already embedded, with the additional advantage of face-selective functionalization provided by the cone shape. The intrinsic cone-shaped geometry of permethlyated α-CD was able to induce kinetic asymmetry. The mechanospecific reaction enhanced directionality and the potential energy stored in the system, which could potentially be used to achieve higher efficiency for long-distance translational motion at the molecular scale.

### 2.7. Catalysis-Driven Active Transport Across a Membrane

**Figure 11:**
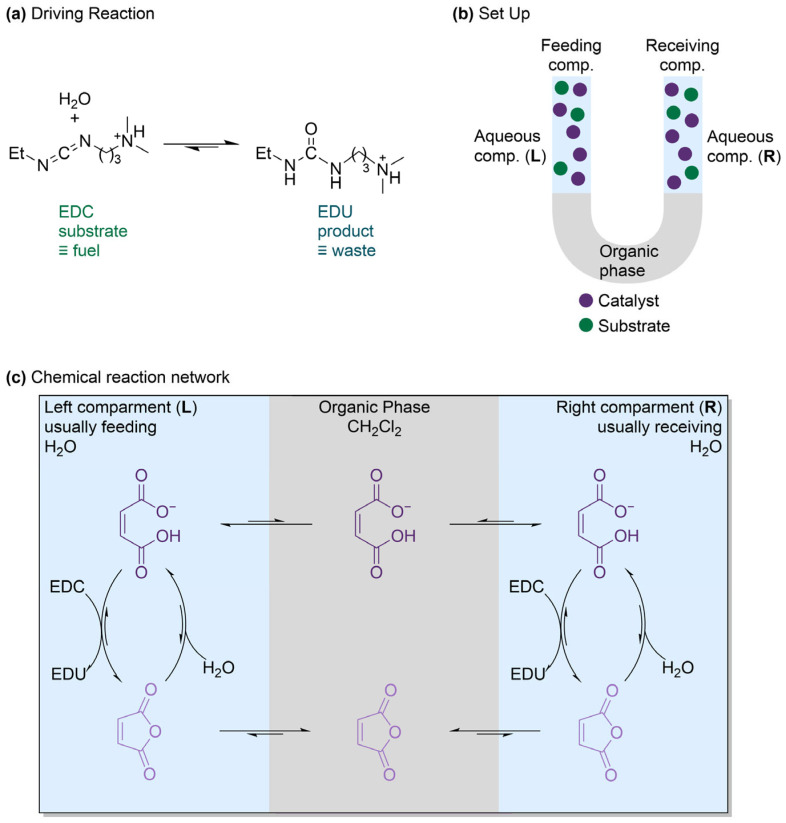
(**a**) The fuel-to-waste reaction used to provide energy and drive active transport when catalyzed by maleic acid. The fuel, 1-ethyl-3-(3-dimethylaminopropyl)carbodiimide (EDC), is converted to waste, 1-ethyl-3-(3-dimethylaminopropyl)urea (EDU). (**b**) Schematic representation of the experimental U-tube setup. The aqueous phase on the left is the feeding compartment (L), while the right compartment is the receiver (R). The two aqueous phases are separated by the organic phase. All three phases were stirred simultaneously using mechanical stirrers (top phases) and a magnetic stirrer (bottom/organic phase). (**c**) The chemical reaction network, showing the compounds across the three phases.

Liang et al. applied the principles of molecular ratchets from small-molecule machines to a transmembrane setting [34]. The few examples of transmembrane primary active transport in artificial systems reported in the literature are light-driven, but the majority of nature’s active transport processes are chemically powered (e.g., the catalysis of ATP hydrolysis transduces energy into a transmembrane gradient). This discrepancy compelled the authors to develop an artificial catalysis-driven system that uses chemical energy from carbodiimide hydration (Figure 11a) to actively transport maleic acid across a hydrophobic liquid membrane, which separates two aqueous compartments. The catalysis-driven transport of maleic acid across the hydrophobic liquid membrane was conducted in a U-tube setup (Figure 11b).

A dichloromethane liquid phase was used as a macroscopic liquid membrane between two 2-(N-morpholino)ethanesulfonic acid (MES) buffered aqueous compartments, with compartment L acting as the feeding compartment and compartment R as the receiver. All three phases were stirred simultaneously, ensuring efficient mixing of phase boundaries. Over a 3 h course, a slight decrease in maleic acid concentration was observed in the L compartment, while no maleic acid was observed in compartment R. Although maleic acid could partially dissolve in the hydrophobic phase, the results suggested that it did not significantly diffuse between the two aqueous compartments throughout the duration of the experiment. Overall, the chemical energy from carbodiimide hydration was transduced and stored as energy as a chemical gradient of maleic acid across a macroscopic liquid membrane. The thermodynamic efficiency of active transport over time was estimated to reach a maximum at approximately 0.35%, compared to a maximum theoretical efficiency of 3.8% for pumping all the maleic acid from L to R using EDC. The theoretical maximum efficiency could even be lowered further to less than 1.8% when considering that the system’s design was designed to have a statistical 50% chance of anhydride transfer from the organic to the receiving aqueous phase. The low efficiency was thought to be caused by the highly exergonic hydration of EDC driving a purely entropic energy storage process. Nevertheless, the system managed to achieve a significant efficiency increase when compared to most systems driven by a carbodiimide hydration. Kinetic asymmetry was achieved by controlling the relative rates of anhydride formation and hydrolysis of maleic acid cargo on either side of the macroscopic liquid membrane. The team demonstrated that a catalysis-driven information ratchet mechanism (Figure 11c) does not only concern molecular motors and pumps, but is also the foundation of transmembrane active transport. Kinetic modeling showed that rate constants measured in isolated systems can be combined to describe and predict the behavior of a full model of the reaction network. In addition, the results pointed out the link between nonlinear phenomena (e.g., autocatalysis and negative feedback) and ratchet mechanisms.

### 2.8. Another U-Tube Setup for Chemically Fueled Active Transport

**Figure 12:**
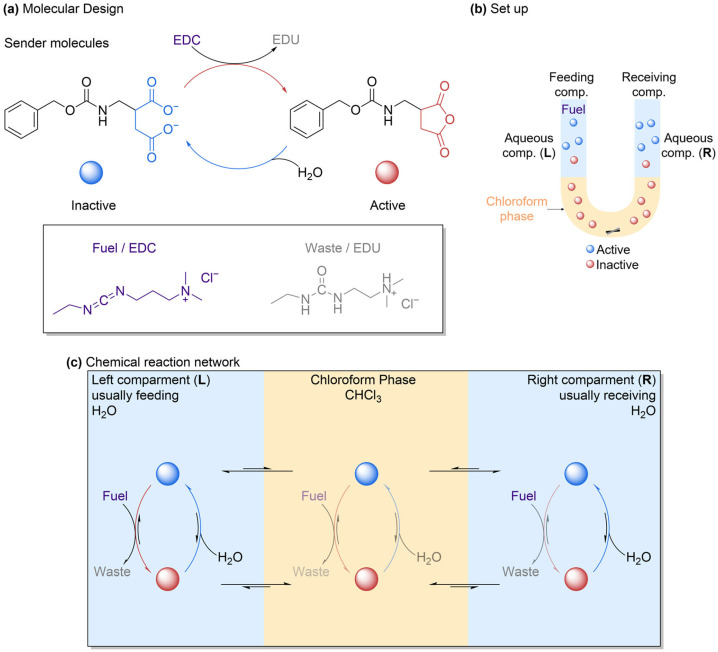
(**a**) Schematic representation of the molecular structure of the sender molecule, anionic-charged dicarboxylate (Cbz-D), its anhydride counterpart, fuel 1-ethyl-3-dimethylaminopropyl carbodiimide (EDC), and waste 1-[3-(dimethylamino)propyl]-3-ethylurea (EDU). (**b**) Schematic representation of the experimental U-tube setup. The aqueous phase on the left is the sender (L), while the right compartment is the receiver (R). The two aqueous phases are separated by the CHCl_3_ phase. A stirring bar stirs the chloroform phase to ensure that the compartment is well mixed. (**c**) The reaction network of the chemically fueled active transport, showing the compounds across the three phases. Fuel activates the sender molecule Cbz-D (blue ball), converting it to the anhydride (red ball). Anhydride and acid Cbz-D partition into the chloroform, travel through it, and leave it at the receiver side. The anhydride partitioning is higher compared to the acid Cbz-D. At the receiver side, anhydride deactivates via hydrolysis. Kinetic asymmetry is what allows active transport of the sender molecule acid Cbz-D.

Kriebisch et al. recently reported a synthetic system to achieve small aqueous transport between two aqueous phases via a centimeter-sized immiscible solvent [35]. While Liang et al. created a U-tube setup featuring CH_2_Cl_2_ as the organic phase that separates the two aqueous phases [34], the U-tube system used by Kriebisch et al. features a chloroform domain as the mediator that separates the aqueous ones (Figure 12).

A stirrer ensured homogeneous mixing of the chloroform phase. The setup was inspired by ATPase, a natural pump that is able to transport molecules across membranes against concentration gradients [36]. In order to achieve directed transport from the sender phase to the receiver phase, EDC (1-ethyl-3-dimethylaminopropyl carbodiimide) was used as fuel such that a sender molecule, anionic-charged dicarboxylate (Cbz-D), could become chemically activated, allowing it to partition in the chloroform barrier and deactivate in the receiver phase without being able to return back. Chemical conversion was thought to lower the kinetic barrier to transport molecules from the sender phase without affecting the barrier from the receiver phase. Directionality was thus established when molecules passed the chloroform barrier. The activation rate was determined by the difference in fuel concentration in the sender and receiver compartments, thus introducing kinetic and diffusion asymmetries to the reaction network. A reaction-diffusion model was used to identify the critical parameters that determined the efficiency of active transport. An efficiency of 100% implies that each molecule of EDC transported one transporter molecule to the sender phase. The reported transport efficiency was 0.25% ± 0.05% when using 100 mM EDC, meaning that it takes 400 molecules of EDC to transport one molecule of Cbz-D from the sender to the receiver. Results showed that minimizing the length of the aqueous compartment was crucial because only a small fraction of the volume close to the chloroform interface in the sender phase contributes to active transport. This observation was experimentally demonstrated by decreasing the sender’s length from 25.4 mm to 3.5 mm, leading to an increase in efficiency by 10-fold to 2.5 ± 0.01 (instead of 400, it takes 40 fuel molecules to transport one Cbz-D molecule to the receiver). This improvement was consistent with the reaction-diffusion model. The proposed mechanism for active transport was considered to bear the key characteristics of an information ratchet. The experiments and model showed that active transport is achievable without external pumps, but such a system works efficiently only if the small molecules are activated close to the phase boundary, have a long lifetime, and can rapidly transfer across the interface and efficiently partition between the aqueous and chloroform compartments. The authors hypothesized that these constraints might be part of the reason why nature evolved more complex pump machinery instead of relying on a simpler setup. While the artificial system is not as complex as its natural inspirations, its design principles could still offer new means of artificial transport across membranes for future applications, such as cargo transport in material and medical applications.

## 3. Discussion

The field of artificial molecular machinery has undergone a profound paradigm shift, moving from the verification of fundamental physical principles to the realization of autonomous, work-performing systems. At the heart of this evolution lies the information ratchet, a mechanism that—much like the conceptual Maxwell’s Demon—rectifies stochastic Brownian motion into directional work by utilizing information about the system’s state [14,37,38]. While early iterations relied on simple positional data of a macrocycle along a track, the recent literature (2023–2025) reveals a move toward structural and kinetic complexity that significantly enhances efficiency and autonomy.

A primary advancement, as demonstrated by Liu et al., is the introduction of cone-shaped macrocycles, specifically functionalized cyclodextrins [30]. In fact, traditional information ratchets typically monitor only the relative position of the components. However, by employing a non-symmetrical, 3D structure, these systems now exploit orientation information. The “cone” shape acts as a steric and electronic filter: the interaction with the axle depends not just on where the macrocycle is, but on which way it is facing. This “shape-dependent” ratcheting allows for a more sophisticated modulation of energy landscapes, effectively doubling the informational input and leading to a more robust selection of the desired kinetic path.

The quest for perfect unidirectionality—a hallmark of biological motors—has seen a breakthrough with the development of double-gated one-way ratchets [32]. Unlike previous models, where some degree of “backsliding” was inevitable due to thermal fluctuations, these new designs utilize the macrocycle itself to actively open and close kinetic gates. In these rotaxanes, the cyclodextrin not only passes through a barrier; it actively facilitates the “opening” of the gate in the forward direction, while remaining blocked in the reverse. This intramolecular “irreversible” mechanism represents a transition from passive ratcheting to active gating, mimicking the sophisticated behavior found in kinesin or dynein.

In ‘Reaction: Of myths, misconceptions, and motors—A matter of equilibrium’, Leigh clarifies why these systems are so effective [39]. The efficiency of a catalysis-driven motor is governed by kinetic asymmetry—the ratio of forward to backward rate constants under non-equilibrium conditions. In a catalytic cycle of a fuel-to-waste reaction, the catalyst’s rotational arcs must differ between two states, such that they can cover the full 360° rotation of the motor, although neither state can individually allow a complete rotation [39]. In order to directionally bias the motor’s rotation, anisotropy is introduced by using chiral fuel, a chiral promoter, or by taking advantage of the chirality in the motor molecule. For instance, the dual-motor designed by Borsley et al. featured a chiral anhydride hydrolysis promoter [24]. By using chemical fuels (e.g., carbodiimides or ATP analogues), these ratchets maintain a high chemical potential gradient. Research now focuses on how to minimize “wasteful” fuel consumption by ensuring that every catalytic event is tightly coupled to a mechanical step. The latest data suggest that by optimizing the structural fit between the track and the motor (the “docking” precision), artificial motors can achieve power outputs that begin to rival their biological counterparts.

Finally, the transition from molecular motion to macroscopic function is exemplified by the work of Kriebisch et al. [35]. These principles are no longer confined to rotaxanes in solution but are applied to active transport across membranes. By using chemically fueled transporters that are transiently activated, molecules can be moved against a concentration gradient across hydrophobic barriers. This demonstrates how information ratchets can play the role of the fundamental “engines” required for systems chemistry to achieve homeostatic control and signal transduction in synthetic cells.

## 4. Conclusions

The recent advancements summarized in this review highlight that artificial information ratchets have moved beyond mere laboratory curiosities. This review was also intended to show the variety of synthetic information ratchets, from single and dual motors to cone-shaped macrocycles and active transport systems across different phases. The integration of 3D molecular geometry (the cone-shape effect) and active kinetic gating has solved the long-standing problem of achieving high directional persistence in the presence of Brownian noise. Researchers are now able to design molecular machines where the “decision” to move forward is hardcoded into the structural and chemical complementarity of the components. As design principles are solidified and new artificial designs are introduced, current molecular machinery can be better understood and optimized.

The implications for the future are at least twofold. First, the ability to perform active transport against gradients opens the door to truly biomimetic materials—synthetic systems capable of autonomous waste removal, nutrient uptake, and self-repair. Second, the development of structure-performance scaling laws (as proposed by the Leigh group) provides a roadmap for engineers to transition from single-molecule motors to cooperative arrays capable of performing macroscopic work, such as the contraction of molecular actuators or the pumping of fluids in microfluidic devices.

Despite the great achievements made, several challenges remain, such as the observation that even if current fuels are effective, they often produce by-products that must be managed. The next generation of information ratchets will likely focus on “cleaner” fueling cycles, perhaps utilizing light or pH gradients, to drive these sophisticated kinetic networks. In addition, as another ambitious attempt to mimic nature’s complicated means, future research may take artificial information ratcheting one step further and aim for the integration of small-molecule machines into wider systems, featuring the compartmentalization and the cooperation via a multiple motor-operation [4,17]. Ultimately, the mastery of the information ratchet mechanism should bring us one step closer to bridging the gap between inanimate matter and the functional complexity of living organisms.

## Data Availability

The data contained within this review are available by accessing the articles cited in the bibliography. No new data were created for this review.
